# Improving Geldanamycin Production in *Streptomyces geldanamycininus* Through UV Mutagenesis of Protoplast

**DOI:** 10.3390/microorganisms13010186

**Published:** 2025-01-17

**Authors:** Yuan Yuan, Lu Yang, Zhikai Fang, Haimin Chen, Fei Sun, Hong Jiang, Jian Zhou

**Affiliations:** 1 Fujian Key Laboratory of Screening for Novel Microbial Products, Fujian Institute of Microbiology, 25 Jinbu Road, Fuzhou 350007, China; m18388376609@163.com (Y.Y.); 13674320840@163.com (L.Y.); fangzk1986@126.com (Z.F.); c363784591@163.com (H.C.); sunfei19811109@163.com (F.S.); 2 The School of Pharmacy, Fujian Medical University, 1 North Xuefu Road, Fuzhou 350122, China

**Keywords:** whole-genome sequencing, transcriptome sequencing, protoplast mutagenesis, UV mutagenesis, geldanamycin

## Abstract

Geldanamycin, a benzoquinone ansa antibiotic, has been extensively applied in medical, agricultural, and health research areas due to its antitumor, antifungal, herbicidal, and antiradiation effects. In this study, an improvement of geldanamycin production by *Streptomyces geldanamycininus* FIM18-0592 was first performed by protoplasts combined with UV mutagenesis and ribosome engineering technology, respectively. The results showed that strains induced by UV mutagenesis of protoplasts were superior to protoplasts treated with erythromycin in terms of the positive variability, average relative titer, and maximum relative titer, with values of 51.95%, 99%, and 136%, respectively. A mutant strain that produced 3742 μg/mL geldanamycin was generated by protoplast UV mutagenesis, with a 36% higher yield than the initial strain. Multi-omic analysis revealed that the high-yielding geldanamycin in mutant strain 53 could upregulate *GdmG* and *GdmX* by 1.59 and 2.38 times in the ansamycin synthesis pathway, and downregulate *pks12*, *pikAI*, and *pikAII* by 0.25, 0.37, and 0.48 times in the fatty acid synthesis pathway, which was crucial for geldanamycin production. Our study provides a novel *S. geldanamycininus* geldanamycin production strategy and offers valuable insights for mutagenesis and breeding of other microorganisms.

## 1. Introduction

Geldanamycin (GA) is a kind of benzoquinone ansa antibiotic derived from *Streptomyces hygrophilus* [1]. It was identified as the first N-terminal inhibitor of heat shock protein 90 (Hsp90) with particularly significant antitumor effects, achieved by specifically inhibiting the ATP/ADP domain of Hsp90 and downregulating a variety of Hsp90 target proteins [2]. At present, numerous geldanamycin derivatives have progressed to the clinical stages as anticancer agents, including tanespimycin (17-AAG), alvespimycin (17-DMAG), ganetespib (STA-9090), and zelavespib (PU-H71) [3]. These derivatives also demonstrate considerable biological activities and physiological functions such as anti-parasite [4], antiviral [5], and antifungal [6] actions. In addition, geldanamycin is important in agriculture, mainly thanks to its highly effective resistance to stresses like radiation, crop pathogens, and weeds [7,8,9,10]. Meanwhile, studies have shown that geldanamycin exerts toxic effects on plants, affecting their morphological characteristics and inhibiting root growth [11,12]. Therefore, geldanamycin can also be utilized to establish plant disease models, which is beneficial to agricultural research. However, there are some problems including low yields and genetic instability in wild-type geldanamycin-producing bacteria, so it is essential to improve the characteristics of strains by applying strain improvement technology.

Recently, multiple strategies have been applied to enhance the production of natural microbial products, mainly CRISPR/Cas9-mediated genetic modification, fermentation optimization, and genetic improvement [13]. Meng et al. found that heterologous expression of nitrite-specific transporter gene *nirC* (*nirC-ABC2* cassette) in *Streptomyces hygroscopicus* XM201 resulted in an increase in the production of geldanamycin from 1590 to 1780 μg/mL [14]. In our previous studies, the fermentation process of *Streptomyces geldanamycininus* FIM18-0592 was optimized and the fermentation titer of geldanamycin reached 2887 μg/mL, which was 66% higher than the original fermentation process [15]. Dobson LF et al. found that a 70% reduction in mycelium diameter resulted in an 88% increase in geldanamycin production, but the fermentation production of geldanamycin is relatively low and insufficient to meet industrial demands [16]. Notably, genetic improvement technology has an important function in improving the quality of industrial strains [17,18]. As for random mutagenesis techniques, UV irradiation is a traditional and successful mutagenesis method, which induces a broad spectrum of point mutations [19]. Meanwhile, in the field of ribosome engineering technology, ribosome-associated antibiotics such as erythromycin, gentamicin, and neomycin can modify the ribosome structure of microorganisms through resistance mutation technology, thereby directly or indirectly activating secondary metabolism and enhancing the production of secondary metabolites [20,21,22,23]. However, there are lots of disadvantages such as a low mutation frequency, few mutation sites, and genetic instability in traditional strain improvement techniques. In addition to the random mutagenesis techniques, protoplast mutagenesis technology can improve the sensitivity of strains to various external mutagenesis conditions by removing the cell wall, and can significantly increase the mutation frequency by combining with other mutagenesis methods, which is conducive to the screening of high-yielding strains [24]. For example, a hyper-producing strain with a 100-fold higher yield than the wild strain was obtained by combining protoplasts with nitrosoguanidine (NTG) mutation and UV irradiation [25]. Genome shuffling combined with ribosome engineering significantly improved virginiamycin production by 11.6-fold compared to the wild-type strain [26].

With the development of biotechnology, especially the next-generation and third-generation sequencing technologies, an increasing number of potentially valuable mutations have been identified [27]. To further investigate these mutations, a deeper understanding of the relationship between genomic mutations and transcriptional changes is crucial [28]. In this study, protoplast mutagenesis combined with UV mutagenesis and ribosome engineering technology, respectively, was used for the first time to enhance geldanamycin production in *S. geldanamycininus* FIM18-0592. Whole-genome sequencing and transcriptome sequencing were subsequently performed to examine the genomic mutations and transcript levels between the original and high-yielding strains. Additionally, the significant differentially expressed genes (DEGs) responsible for geldanamycin synthesis were identified by qRT-PCR. These results could lay the foundation for the development and application of geldanamycin products.

## 2. Materials and Methods

### 2.1. Strain and Growth Conditions

*S. geldanamycininus* FIM18-0592 was isolated in our laboratory and deposited as a patented strain at the China General Microbial Strain Preservation Management Center (CGMCC, Beijing, China) with the number NO.: 27634. *S. geldanamycininus* was cultivated in a 250 mL flask containing 30 mL seed medium (glucose 18 g/L, maltodextrin 10 g/L, soybean cake powder 22.5 g/L, MgSO_4_·7H_2_O 1 g/L, K_2_HPO_4_·3H_2_O 1 g/L, pH 7.2) and incubated at 28 °C, 240 rpm for 48 h. Then, the seed was transferred to the fermentation medium (glucose 104.2 g/L, soybean cake powder 16.8 g/L, ammonium sulfate 3 g/L, lactic acid 3 g/L, glycerin 40 g/L, magnesium sulfate 1 g/L, calcium carbonate 4 g/L, pH 7.2) at 5% inoculum and incubated at 28 °C, 240 rpm for 120 h [15]. Finally, the mycelia were collected at 4 °C, 12,000 rpm for 3 min to determine the biomass, then rapidly frozen in liquid nitrogen for 15 min and subsequently stored at −80 °C.

### 2.2. Preparation and Optimization of Protoplasts

The primary seed of *S. geldanamycininus* FIM18-0592 was inoculated on soybean-casein digest medium (TSB) (tryptone 17 g/L, soy peptone 3 g/L, glucose 2.5 g/L, sodium chloride 5 g/L, K_2_HPO_4_·3H_2_O 2.5 g/L, pH 7.3) at 28 °C, 280 rpm for 48 h. Under the same culture conditions, 6.5% primary seed in the logarithmic growth stage was inoculated in TSB medium containing glycine for 24 h. The mycelia from the fermentation broth were dispersed with ultrasound and harvested at 4000 rpm for 10 min. The collected mycelia were washed twice with 10.3% sucrose and P buffer (sucrose 128.75 g/L, K_2_SO_4_ 0.31 g/L, MgCl_2_·6H_2_O 2.53 g/L, trace element solution 1.25 mL per liter) at 4000 rpm for 10 min, respectively. The reaction solution of mycelia and lysozyme was incubated with agitation at 35 °C for 80 min, and the formation of protoplasts in the lysis solution was observed every 20 min under an optical microscope. After enzymolysis, the protoplast suspension was filtered through filter paper and centrifuged at 1000× *g* rpm for 5 min to obtain a precipitate. The precipitate was washed twice with P buffer at 1000 rpm for 5 min, and the harvested protoplasts were resuspended in P buffer [29].

Influencing factors of protoplasmic preparation were independently investigated and optimized, including mycelial growth conditions, enzymatic time, enzyme concentration, glycine concentration, ultrasonic time, and sucrose concentration. During cultivation of the seed solution, the biomass was measured every 2 h and growth curves were plotted. The enzymatic hydrolysis time was optimized from 10 to 70 min. The enzyme concentrations were optimized from 20,000 to 80,000 U/mL. The concentrations of glycine were optimized from 0 to 8 g/L. The ultrasonic time was optimized from 0 to 6 s. The sucrose concentrations in P buffer were optimized from 10.3 to 14%.

### 2.3. Determination of Protoplast Preparation Rate and Regeneration Rate

The mycelial suspension without lysozyme digestion was diluted in sterile water and incubated on ISP2 medium (yeast extract 4 g/L, malt extract 1 g/L, glucose 4 g/L, agar 20 g/L, pH 7.3) at 28 °C for 5 d, and the number of colonies was recorded as A. The protoplast suspension was diluted, respectively, in sterile water and P buffer and incubated on ISP 2 medium and regeneration medium at 28 °C for 5 d, the number of colonies was recorded as B and C, respectively. The preparation rate and regeneration rate of protoplasts were calculated based on the following formulas [30]:

Preparation rate = (A − B)/A × 100%

Regeneration rate = (C − B)/(A − B) × 100%

### 2.4. Mutagenesis of Protoplasts

The prepared protoplast suspension was separately treated with ultraviolet irradiation and erythromycin to improve the mutation rate of the strain. Based on prior studies, the UV mutagenesis of protoplasts was exposed under a 30 W UV lamp at a distance of 30 cm, and 7 irradiation periods of 0 s, 30 s, 60 s, 90 s, 120 s, 150 s, and 180 s were set, respectively [30]. After irradiation, the protoplast suspension was diluted 10 times with P buffer and coated on regenerative medium at 28 °C without light for 5 d, and then the colony morphology was observed and fatality rate calculated. The minimum inhibitory concentration (MIC) of *S. geldanamycininus* FIM18-0592 protoplasts was determined using the agar dilution method [31]. The protoplast suspension was coated on regenerative medium with erythromycin concentrations of 0 μg/mL, 0.5 μg/mL, 1.0 μg/mL, 1.5 μg/mL, 2.0 μg/mL, 2.5 μg/mL, and 5.0 μg/mL, respectively. After culturing, the colony morphology was observed and fatality rate calculated.

### 2.5. Screening of High-Yielding Strains

After treatment with ultraviolet radiation and erythromycin, the colonies were screened under mutagenesis conditions with an 80% fatality rate. A total of 117 colonies were selected based on the features of having different shapes and fast growth in order to obtain mutant strains with high yields of geldanamycin. The genetic stability of these selected high-yielding strains was assessed by examining the relative titers during five passages.

### 2.6. Detection and Quantification of Geldanamycin Titer

The geldanamycin standard solution (Shanghai Yuanye Biotechnology Co., Ltd., Shanghai, China) was prepared and diluted to 1000 μg/mL, 750 μg/mL, 500 μg/mL, 250 μg/mL, and 100 μg/mL using methanol. Subsequently, the peak area was determined by HPLC, and the standard curve was constructed. The fermentation solution was mixed with methanol at a volume ratio of 1:4 and centrifuged at 4000× *g* rpm for 20 min, after ultrasound for 30 min. To fully remove impurities, the supernatant was centrifuged at 10,000× *g* rpm for 20 min. The supernatant was filtered through a 0.22 μL filter into the sample bottle. An Agilent 1260 Infinity II HPLC system was used to determine the geldanamycin titer, DAD detector, column: Agilent Syncronis C18 (4.6 mm × 250 mm, 5 μm), mobile phase: 75% methanol, sample size: 10 μL, flow rate: 1 mL/min, detection wavelength: 304 nm.

### 2.7. DNA Extraction and Whole-Genome Sequencing

The total DNA from the initial strain FIM18-0592 and the high-yielding strain 53 was extracted according to the instructions of the Vazyme Fast Pure Bacteria DNA Kit (Vazyme Biotech Co., Ltd., Nanjing, China). The total quantity and integrity of DNA were determined using the Quant-iT PicoGreen dsDNA Assay Kit (Thermo Fisher Scientific Inc., Waltham, MA, USA) and 1% agarose gel electrophoresis, respectively. Whole-genome sequencing of FIM 18-0592 was performed by Shanghai Personalbio Biotechnology Co., Ltd., Shanghai, China, with both the next-generation sequencing technology provided by the Illumina NovaSeq sequencing platform and the third-generation sequencing technology provided by the PacBio Sequel sequencing platform. The high-yielding strain was next-generation sequenced using FIM18-0592 as the reference genome. High-quality data were obtained by filtering the raw data. The filtered high-quality data were subsequently aligned with the reference genome using the program bwa mem (0.7.12-r1039). The GATK 4.0 software was used to detect SNPs (single-nucleotide polymorphisms) and InDels (insertions and deletions), and SNP and InDel sites were annotated by the ANNOVAR software (https://annovar.openbioinformatics.org/en/latest/, accessed on 17 May 2024).

### 2.8. RNA Extraction and Transcriptome Sequencing

Two mycelia samples from the fermentation broth of the initial strain FIM18-0592 and the high-yielding strain 53 after cultivation for 96 h were immediately collected at 4 °C and 12,000 rpm for 3 min, and the precipitate was centrifuged again to fully remove the supernatant; then, the samples were quickly frozen in liquid nitrogen for 15 min and stored at −80 °C. The total RNA was extracted from the fermentation broth of the initial strain and the high-yielding strain after cultivation for 96 h. The quality and concentration of the extracted total RNA were determined by agarose gel electrophoresis and an Agilent 2100 Bioanalyzer. Prokaryotic transcriptome sequencing was performed by Shanghai Personalbio Biotechnology Co., Ltd. with the Illuminate NovaSeq 6000 platform.

### 2.9. Transcriptome Data Analysis

After transcriptome sequencing was performed, approximately 8 GB of data was obtained from the Illumina NovaSeq 6000 platform. The raw data were filtered to remove the reads connecting poly-N and low-quality reads, to harvest clean reads, and the filtered sequences were compared with the corresponding reference genome (FIM18-0592) in terms of reads’ mapped ratio, area distribution, and depth distribution. HTSeq 0.6.1p2 was used to calculate the gene expression levels, and these were further normalized by fragments per kilobase million (FPKM). Differentially expressed genes (DEGs) were identified between the initial strain and the high-yielding strain based on a fold change of |log2FoldChange| > 1 and a significance level of *p* < 0.05. Multiple in-depth studies were carried out, including gene ontology (GO) function analysis, https://www.geneontology.org/ (accessed on 17 May 2024), Kyoto Encyclopedia of Genes and Genomes (KEGG) pathway analysis, https://www.genome.jp/kegg/ (accessed on 17 May 2024), correlation analysis of DEGs, and so on [32]. The interesting DEGs with extremely significant changes in the geldanamycin synthesis pathway were selected for validation, and the main pathways included the ansamycin synthesis pathway, glycolysis pathway (EMP), pantothenate and CoA biosynthesis pathway, citrate cycle (TCA cycle), valine, leucine, and isoleucine degradation pathway, pyruvate metabolism, fatty acid biosynthesis pathway, and pentose phosphate pathway (PPP). The interactions of interesting DEGs in the synthetic cluster of geldanamycin were visualized by Cytoscape 3.7.2. The biosynthetic pathway illustrations of geldanamycin were drawn by Adobe Illustrator 2021.

### 2.10. Reverse Transcription and qRT-PCR Analysis

Based on the transcriptome sequencing results, 16 differentially expressed genes affecting the production of geldanamycin were selected for qRT-PCR to verify the reliability of transcriptome sequencing. The primers were designed by Primer 6.0 and synthesized by Tsingke Biotechnology Co., Ltd., Xiamen, China. The sequences of these primers are shown in Table 1. The total RNA from the initial strain and the high-yielding strain after liquid fermentation for 96 h was extracted with a Trizol kit (Sangon, Shanghai, China). Residual genomic DNA was removed with 5xgDNA digester mix, and the RNA was reverse-transcribed into cDNA with 4xIIIM-MLV RT Mix (Sangon, Shanghai, China). The qRT-PCR experiment was performed with SGExcel FastSYBR qPCR Mix (Sangon, Shanghai, China) in an SLAN-96H real-time PCR system (Hongshi, Shanghai, China) to compare the transcription levels of differentially expressed genes.

### 2.11. Statistical Analysis

SPSS 25.0 statistical software (IBM Co., Armonk, NY, USA) was used to determine the statistical significance of differences among groups. Data were expressed as the mean ± standard deviation (*x* ± *s*), and the *t*-test was used for inter-group comparison. *p* < 0.05 meant a significant difference, and *p* < 0.01 meant an extremely significant difference.

## 3. Results

### 3.1. Effects of Various Factors on the Preparation Rate and Regeneration Rate of FIM18-0592 Protoplasts

Protoplast mutagenesis technology serves as an effective mutation technique with distinct advantages in microbial strain improvement, which can integrate physical and chemical mutagens to elevate the mutation frequencies and further improve the compound yield [33]. For example, a ursodeoxycholic acid-producing *Gibberella zeae* M23 strain with a 1.26-fold higher yield than wild strains was obtained by combining protoplasts with UV mutagenesis [34]. To improve the fermentation yield of geldanamycin, we sought to screen high-yielding strains to shorten the fermentation time and reduce the fermentation cost for large-scale fermentation production. So, protoplasts were combined with UV mutagenesis and ribosome engineering technology, respectively, and we screened for high-yielding mutant strains.

**Figure 1:**
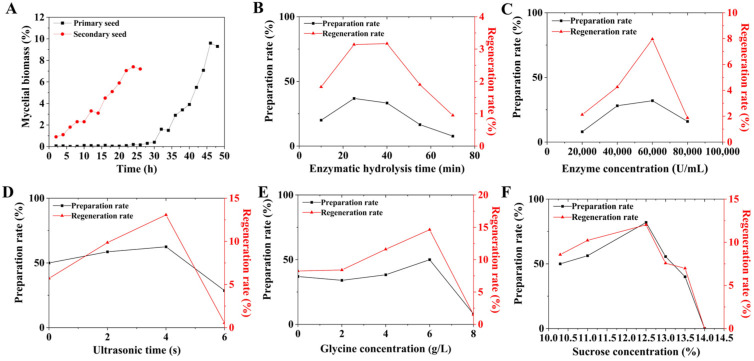
Optimization of the preparation rate and regeneration rate of FIM18-0592 protoplasts. (**A**) The growth curve of FIM18-0592 cultured for 24 h and 48 h; (**B**) effect of enzymatic hydrolysis time on the preparation rate and regeneration rate; (**C**) effect of enzyme concentration on the preparation rate and regeneration rate; (**D**) effect of ultrasonic time on the preparation rate and regeneration rate; (**E**) effect of glycine concentration on the preparation rate and regeneration rate; (**F**) effect of sucrose concentration in the P buffer on the preparation rate and regeneration rate.

As is known, multiple factors such as glycine concentration, ultrasonic time, enzyme concentration, and so on can affect the preparation of protoplasts [29]. Therefore, in order to improve the preparation and regeneration rates of FIM18-0592 protoplasts, these influencing factors were optimized in the culture and preparation process. Studies have demonstrated that the mycelia during the logarithmic growth phase show high protoplast preparation and regeneration rates [30]. So, the primary seed was cultured for 44 h, and the secondary seed for 22 h (Figure 1A). In the process of enzymatic hydrolysis, both the preparation rate and regeneration rate of protoplasts gradually increased with the increase in lysozyme concentrations and enzymatic hydrolysis time. However, too high a lysozyme concentration and too long an enzymatic hydrolysis time will adversely affect the preparation and regeneration of protoplasts, because complete hydrolysis of the cell wall can significantly decrease the protoplast activity. Accordingly, when the enzyme concentration was 60,000 U/mL, enzymatic hydrolysis for 25 min was most appropriate (Figure 1B,C). Ultrasound can promote the release of protoplasts by dispersing mycelia and increasing the contact surface between the mycelia and the lysozyme, and a 4 s ultrasonic treatment was most suitable in this regard (Figure 1D). Glycine can facilitate the release of protoplasts by inhibiting the synthesis of the cell wall, and a concentration of 6 g/L was optimal from this perspective (Figure 1E). An appropriate sucrose concentration is crucial for balancing the internal and external osmotic pressures of protoplasts, which is conducive to maintaining the number of protoplasts and the binding of enzymes and substrates. At a sucrose concentration of 12.5% in the P buffer, the protoplast preparation rate was as high as 82% (Figure 1F), indicating that FIM18-0592 was suitable for preparing protoplasts under these conditions.

### 3.2. Analysis of the Mutagenesis Results for FIM 18-0592 Protoplasts

**Figure 2:**
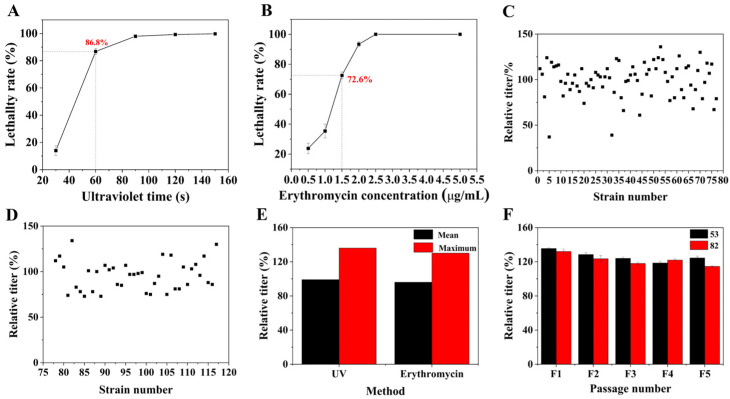
Screening of high-yielding strains by protoplast mutagenesis. (**A**) The lethal curve of protoplast UV mutagenesis. (**B**) The lethal curve of protoplast erythromycin resistance. (**C**) Screening results of protoplast UV mutagenesis. (**D**) Screening results of protoplast erythromycin resistance. (**E**) Comparative analysis of the relative titers across different methods. (**F**) Genetic stability assessment among high-yielding strains.

Protoplasts are sensitive to external factors as they have no cell wall [24]. Therefore, FIM 18-0592 protoplasts were, respectively, mutated by UV irradiation and erythromycin to introduce mutation sites. The generation of protoplasts with forward mutations occurred when the lethality rate was approximately 70–80% [35]. With the extension of ultraviolet irradiation time, the lethality rate of protoplasts rapidly increased and then plateaued (Figure 2A). The lethality rate was 86.8% after 60 s of ultraviolet irradiation, and 77 strains were screened by flask fermentation culturing in this condition. The experimental results showed that the positive variability of UV mutation was as high as 51.95%, the negative variability was 46.75%, and the yield of strain 53 increased by 36% (Figure 2C). We also investigated the lethality of FIM 18-0592 protoplasts at different erythromycin concentrations. With increasing concentration of erythromycin, the lethality of protoplasts initially increased and then leveled off (Figure 2B). The lethality rate was 72.6% when the concentration of erythromycin was 1.5 μg/mL, and 40 strains were screened under this condition. The positive variability was 40%, the negative variability was 57.5%, and the yield of strain 82 increased by 30% (Figure 2D). At the same time, UV mutagenesis of protoplasts was superior to protoplasts treated with erythromycin in both average and maximum relative titers, with values of 99% and 136% (Figure 2E). We also explored the genetic stability of the two high-yielding strains by examining the relative titers during five passages; the average relative titers of strains 53 and 82 were 133.67 ± 3.21% and 130 ± 4%, respectively, and the relative titer of strain 53 was more stable than that of strain 82 (Figure 2F). In sum, strain 53 obtained by UV mutagenesis of protoplasts demonstrated a higher titer and better genetic stability, indicating that it has promising development prospects.

### 3.3. Detection and Quantification of Geldanamycin Between FIM 18-0592 and High-Yielding Strain 53

**Figure 3:**
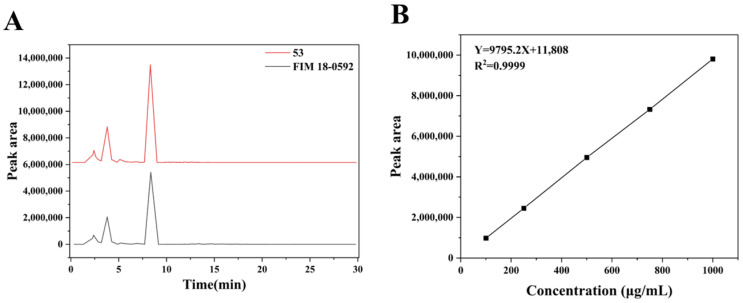
Detection and quantification of geldanamycin by HPLC analysis. (**A**) HPLC spectra of FIM18-0592 and high-yielding strain 53. (**B**) The standard curve of geldanamycin.

There was a significant difference in the peak area of geldanamycin between FIM18-0592 and high-yielding strain 53 (Figure 3A). To quantify the fermentation titer of geldanamycin, a standard curve of the geldanamycin standard was constructed (Figure 3B). The correlation coefficient R^2^ of the standard curve was 0.9999, indicating a strong linear relationship between the peak area and the concentration, and the titer of geldanamycin was calculated using the regression equation X = (Y − 11,808)/9795.2 × 5. In this way, we found the fermentation titers of FIM18-0592 and high-yielding strain 53, respectively, reached 2757 μg/mL and 3742 μg/mL, with a 36% increase in geldanamycin production.

### 3.4. Comparative Analysis of Morphological Characteristics Between FIM 18-0592 and High-Yielding Strain 53

**Figure 4:**
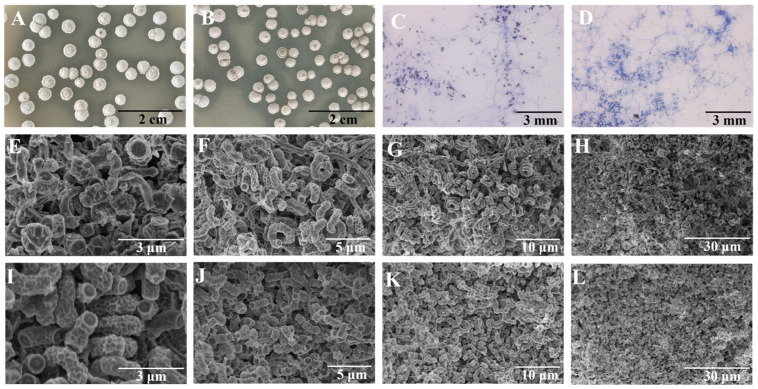
Morphological characteristics of FIM18-0592 and high-yielding strain 53. (**A**) Colony morphology of FIM18-0592. (**B**) Colony morphology of high-yielding strain 53. (**C**) Mycelia morphology of FIM18-0592 (400×). (**D**) Mycelia morphology of high-yielding strain 53 (400×). (**E**–**H**) SEM of FIM18-0592. (**I**–**L**) SEM of high-yielding strain 53.

To explore the distinctions between the high-yielding strain 53 and the original strain, we first compared their morphological characteristics. The dilution coated plate method was used to observe the differences in colony morphology between the high-yielding strain and the original strain. We found that the colonies of the high-yielding strain were fuller and darker than those of the original strain under the same conditions (Figure 4A,B). We also compared mycelia and spores of the high-yielding and original strains. The high-yielding strain possessed more extensive mycelial branches and a greater quantity of spores than the original strain under an optical microscope (Figure 4C,D). Moreover, it was clear that the high-yielding strain had more spores than the original strain under an electron microscope (Figure 4E–L).

### 3.5. Whole-Genome Sequencing Analysis of FIM18-0592

Whole-genome sequencing of FIM 18-0592 was performed to probe the genome profile of the original strain FIM18-0592. The sequencing results showed that the genome length of FIM18-0592 was 12,011,127 bp, with a GC content of 70.75%.

### 3.6. Resequencing Analysis of High-Yielding Strain 53

We further conducted a resequencing analysis on high-yielding strain 53 to elucidate the genomic variation between the original strain and the high-yielding mutant strain. As described in Table 2, SNP and InDel detection were performed on high-yielding strain 53, and the mutation sites were analyzed. SNP detection identified a total count comprising 355 heterozygous SNPs and 1 homozygous SNP (chr-3081). The gene *PpSQ1 00400* (chr-3081) converted its cytosine to thymine with a non-synonymous mutation; it belongs to the leucine, isoleucine, valine, pantothenic acid, and CoA synthesis pathways. InDel detection results revealed three heterozygous InDels and two homozygous InDels (chr-1222 and chr-1226). Both genes *pikAI* (chr-1222) and *pikAII* (chr-1226) belong to fatty acid biosynthesis signaling pathways, with an adenine deletion in *pikAI* (chr-1222) and a cytosine insertion in *pikAII* (chr-1226). Therefore, in order to explain how these pathways affect the production of geldanamycin, we focused on the differential expression of genes within these pathways.

### 3.7. Transcriptome Sequencing Analysis of FIM18-0592 and High-Yielding Strain 53

#### 3.7.1. Analysis of DEGs Between High-Yielding and Original Strains

**Figure 5:**
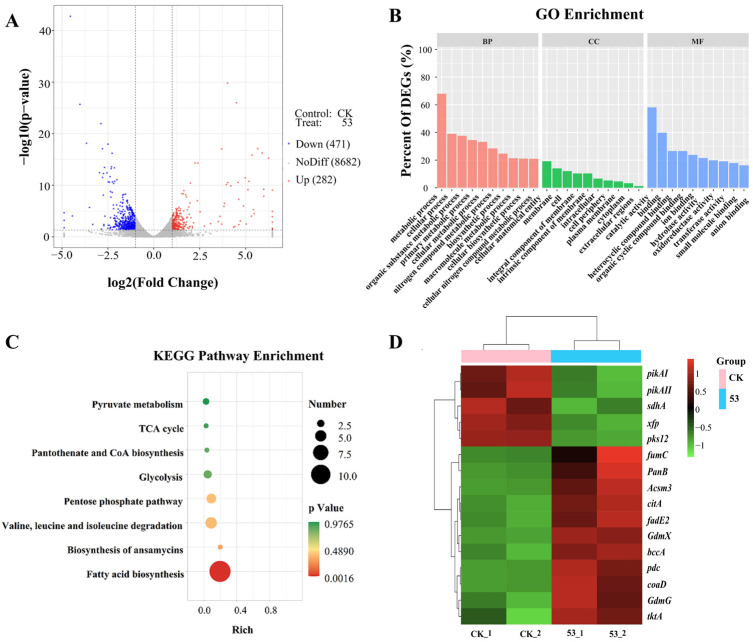
GO function and KEGG pathway analysis of differentially expressed genes. (**A**) Volcano plot analysis of DEGs between FIM18-0592 and high-yielding strain 53. (**B**) GO enrichment analysis of differentially expressed genes. (**C**) KEGG pathway analysis of differentially expressed genes. (**D**) Correlation heat map of differentially expressed genes.

Subsequently, to investigate gene expression differences in the geldanamycin synthetic pathway of the high-yielding strain 53, transcriptome sequencing was conducted to detect the differences in transcript levels between the high-yielding strain 53 and the initial strain. A total of 753 DEGs were detected between the high-yielding and original strains, with 282 DEGs upregulated and 471 DEGs downregulated (Figure 5A). To further identify the biological functions of these DEGs and the related metabolic pathways, GO function enrichment and KEGG pathway enrichment were performed. The GO enrichment of DEGs mainly included cellular components (CCs), biological processes (BPs), and molecular functions (MFs). The DEGs of the high-yielding strain were primarily involved in metabolic processes in the BPs, as well as catalytic activity and binding in the MFs (Figure 5B). These DEGs were mainly involved in the pathways of fatty acid biosynthesis; valine, leucine, and isoleucine degradation; pentose phosphate; and glycolysis (Figure 5C). Furthermore, a correlation heat map was generated based on the expression levels of the interesting genes among the DEGs for the high-yielding and original strains. The result showed that 11 genes were upregulated and 5 genes were downregulated in interesting DEGs (Figure 5D).

#### 3.7.2. Analysis of Metabolic Pathway of Key Genes with Significant Differential Expression

**Figure 6:**
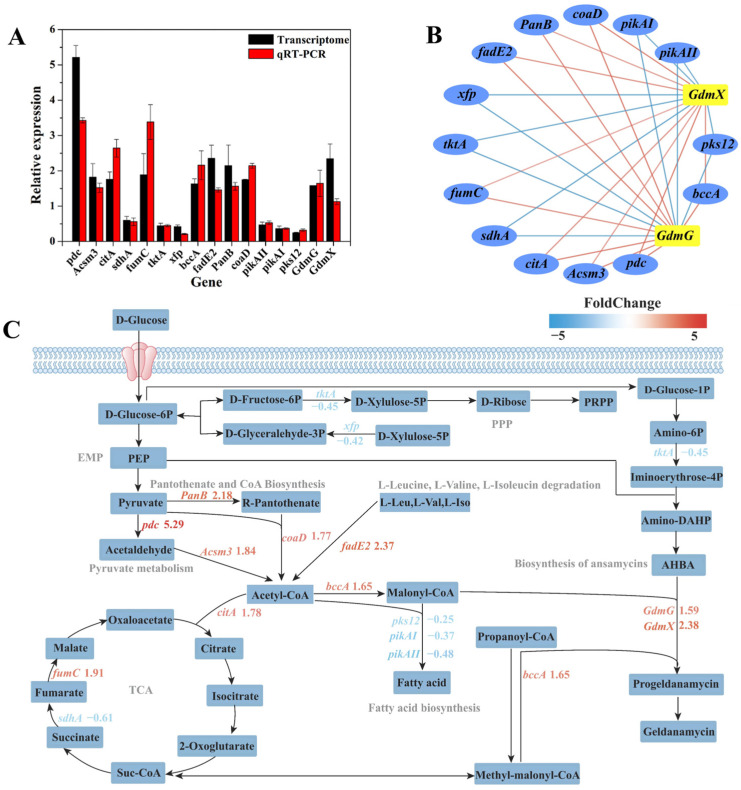
Analysis of key genes with significant differential expression in the geldanamycin metabolic pathways. (**A**) Validation analysis of key genes’ expression levels determined by qRT-PCR. (**B**) Correlation analysis of key genes affecting the yield of geldanamycin. Red lines represent positive correlations, blue lines represent negative correlations, and darker colors indicate a stronger correlation. (**C**) Analysis of key genes in the geldanamycin biosynthetic pathways.

To further verify the reliability of transcriptomic sequencing, qRT-PCR was performed to elucidate the relative expression levels of differentially expressed genes responsible for geldanamycin synthesis. The changes in gene expression levels between the initial strain and high-yielding strain 53 were consistent with the results of the transcriptome sequencing (Figure 6A). The interactions of differentially expressed genes in the synthetic cluster of geldanamycin, including *GdmG*, *GdmX,* and others, were visualized by Cytoscape. *GdmG* and *GdmX* were positively regulated by differential genes such as *pdc*, *Acsm3*, *citA*, *fumC*, *fadE2*, *PanB*, *coaD*, and *bccA* and negatively regulated by *sdhA*, *tktA*, *xfp*, *pikAII*, *pikAI*, and *pks12* (Figure 6B).

We also analyzed the differences in gene expression levels in the geldanamycin synthesis pathway to explore the potential mechanisms for improving geldanamycin yield in *S. geldanamycininus* 53. The biosynthesis of geldanamycin is generally divided into three steps: the biosynthesis of 3-amino-5-hydroxybenzoic acid (AHBA), polyketide synthase (PKS) chain extension, and post-PKS modifications [36]. The AHBA starter unit is synthesized from glucose by the amino shikimate pathway, while the gene cluster *rif*GHJKLMN is necessary for the synthesis of this compound in *Amycolatopsis mediterranei* [37]. The PKS precursor consists of the starting unit AHBA and the expanding units, including 1 malonyl-CoA, 4 methylmalonyl-CoA, and 2 methoxymalonyl-ACP [36]. In general, PKS consists of a group of multifunctional enzymes encoded by a large gene cluster that facilitate the synthesis of geldanamycin [38]. Notably, the synthetic gene cluster of geldanamycin was first identified in *Streptomyces hygroscopicus* NRRL 3602 by Allen and Ritchie [39]. Rascher et al. annotated the gene cluster of geldanamycin in *Streptomyces hygroscopicus* NRRL 3602. *GdmA1*, *GdmA2*, and *GdmA3* are responsible for PKS synthesis, *GdmH*, *GdmI*, *GdmJ*, *GdmK*, and *GdmG* are responsible for the synthesis of glycolate units on PKS, and *GdmL*, *GdmM*, *GdmN*, *GdmX*, and *GdmP* are implicated in postmodification, while *GdmO* is associated with AHBA synthesis [40]. Interestingly, our study found that the expression levels of O-methyl transferase (*GdmG*) and nuclear transport factor 2 family protein (*GdmX*) in the ansamycin synthesis pathway were, respectively, upregulated by 1.59 and 2.38 times in the high-yielding strain 53, especially the function of *GdmG* in methoxy-malonyl-ACP biosynthesis, which was conducive to the synthesis of progeldanamycin (Figure 6C). Similarly, Wang et al. reported that the upregulation of the PKS genes *GdmA1* and *GdmA3* through a strong endogenous promoter resulted in an increased production of geldanamycin, from 773 μg/mL of the initial strain to 1450 μg/mL of the derived strain [36].

Malonyl-CoA is a common precursor of fatty acid and geldanamycin synthesis, suggesting that weakening the fatty acid synthesis pathway may be beneficial to increase geldanamycin yield [41]. Surprisingly, most genes in the fatty acid biosynthesis pathway were significantly downregulated in the high-yielding strain 53, such as beta-ketoacyl synthase (*pks12*), type I polyketide synthase (*pikAI*), and type I polyketide synthase (*pikAII*), which were downregulated by 0.25, 0.37, and 0.48 times, further complementing the resequencing results (Figure 6C). As is well-known, the polyunsaturated fatty acids (PUFAs) are mainly synthesized through the PKS pathway in some microorganisms [42,43]. Therefore, the downregulation of *pks12*, *pikAI*, and *pikAII* may inhibit the synthesis of fatty acids, which was conducive to further synthesis of progeldanamycin by malonyl-CoA. Meanwhile, acetyl-CoA carboxylase (ACCase) catalyzes the conversion of acetyl-CoA to malonyl-CoA [44], indicating that increasing acetyl-CoA can enhance geldanamycin production. Acetyl-CoA is an important metabolic intermediate in the catabolism of carbohydrates, lipids, and amino acids [45]. The results showed that the genes encoding pyruvate decarboxylase (*pdc*) and acyl-CoA synthetase (*Acsm3*) in the EMP pathway were upregulated by 5.29 and 1.84 times, which can promote acetyl-CoA production to promote the synthesis of progeldanamycin. Similarly, the genes encoding 3-methyl-2-oxobutanoate hydroxymethyltransferase (*PanB*) and pantetheine-phosphate adenylyltransferase (*coaD*) in the pantothenate and CoA biosynthesis pathway were upregulated by 2.18 and 1.77 times, which was favorable for the synthesis of acetyl-CoA. The genes encoding acyl-CoA dehydrogenase (*fadE2*) and biotin carboxylase N-terminal domain-containing protein (*bccA*) in the valine, leucine, and isoleucine degradation pathway were upregulated by 2.37 and 1.65 times, which can promote the synthesis of acyl-CoA and malony-CoA, respectively. At the same time, the genes encoding citrate synthase (*citA*) and fumarate hydratase (*fumC*) in the TCA cycle were upregulated by 1.78 and 1.91 times, while succinate dehydrogenase (*sdhA*) was downregulated by 0.61 times, which was beneficial to synthesize methyl-malony-CoA. In addition, the phosphoketolase gene (*xfp*) and transketolase gene (*tktA*) in the PPP were downregulated by 0.42 and 0.45 times, which may promote other metabolic pathways involved in the synthesis of geldanamycin.

Above all, the geldanamycin metabolic pathways of DEGs mainly involved the ansamycin biosynthesis pathway, EMP pathway, pantothenate and CoA biosynthesis pathway, valine, leucine and isoleucine degradation pathway, pyruvate metabolism, TCA cycle, fatty acid synthesis pathway, and PPP (Figure 6C). Among these pathways, the upregulation of the EMP pathway, pantothenate and CoA biosynthesis pathway, valine, leucine and isoleucine degradation pathway, and pyruvate metabolism, and a significant downregulation of the fatty acid synthesis pathway, may promote the ansamycin biosynthesis pathway in *S. geldanamycininus* 53. Currently, the molecular mechanism of *S. geldanamycininus* 53 is still not fully clear, and further studies should be conducted to confirm the specific effects of the relevant genes on geldanamycin production through genetic knockout or overexpression. The high-yielding strains obtained through UV mutagenesis of protoplasts exhibit high efficiency and genetic stability, which is conducive to large-scale industrial production. However, the fermentation production of these strains is significantly influenced by the cultivation conditions, making stable and appropriate fermentation conditions essential.

## 4. Conclusions

In this study, a high-yielding geldanamycin strain was produced. First, the preparation and regeneration conditions of the protoplasts derived from *S. geldanamycininus* FIM18-0592 were optimized. The optimal conditions for protoplast preparation were a primary seed cultured for 44 h, secondary seed cultured for 22 h in medium containing 6 g/L glycine, and mycelia sonicated for 4 s and treated with lysozyme at 60,000 U/mL under a 12.5% sucrose concentration for 25 min. Under optimized conditions, the protoplast preparation rate reached 82%, while the regeneration rate was 12.02%. Next, a high-yielding strain *S. geldanamycininus* 53 with a 1.36-fold higher yield than the initial strain was obtained through UV mutagenesis of protoplasts, and the fermentation titer reached 3742 μg/mL. Finally, whole-genome and transcriptome analysis revealed that the potential mechanisms for obtaining high-yielding geldanamycin in mutant strain 53 may be the upregulation of *GdmG* and *GdmX,* by 1.59 and 2.38 times, in the ansamycin synthesis pathway and the downregulation of *pks12*, *pikAI,* and *pikAII,* by 0.25, 0.37, and 0.48 times, in the fatty acid synthesis pathway. In summary, this study contributes to better production of geldanamycin and provides new ideas for the regulation of geldanamycin biosynthesis.

## Figures and Tables

**Table 1:** Primers for qRT-PCR.

Order	Name	Gene ID	Primer
1	*pdc*	chr_1968-F1	TGCCAGGAAATCTCCACTCA
		chr_1968-R1	CGTAGAAGTGTCCGTTGTCCA
2	*Acsm3*	chr_107-F1	ATTCACCACCGTGGACACCG
		chr_107-R1	CCGCGCCGTAGAATACGTCT
3	*citA*	chr_4649-F1	TCGTACCCGGACTCGAAGGA
		chr_4649-R1	AGGCACCGTCGACCAGCA
4	*sdhA*	chr_4555-F1	ACAAGGGCAAGCGGTTCA
		chr_4555-R1	TTGTAGTCGAGGCGGATGG
5	*fumC*	chr_6221-F	GCGTGGTGGACAAGGACATC
		chr_6221-R	AGGACTGGCTGGCGTTGAC
6	*tktA*	chr_10051-F	GACCGCAACGACCACTCAC
		chr_10051-R	GAACGCCCGCCACTTGTC
7	*xfp*	chr_8692-F1	AAATGGCTGAAGGTGTCCC
		chr_8692-R1	GTGCGAGAAGCCGTTGTG
8	*bccA*	chr_4470-F1	GTGCTTCGTCGAGCGCTAC
		chr_4470-R1	CCTTGAGGATGGCTTTGGAG
9	*fadE2*	chr_2830-F1	CGTGATGGGCAAGACCG
		chr_2830-R1	CCGTGGTAGTGGTCCTCGTA
10	*PanB*	chr_3428-F	CGCAGAAGCAGTCCGAGAAG
		chr_3428-R	TCGTAGGCGGTGAGCATCG
11	*coaD*	chr_6777-F	CGCCAGACCACCGAGGAG
		chr_6777-R	CTGGAGGAGAGGAAGCTGTAGG
12	*pikAII*	chr_1226-F1	GGGACCTCGACTCGCTCTAT
		chr_1226-R1	GGCGAGATCCCGAAGAAA
13	*pikAI*	chr_1222-F	TGGAGGCGATTCACGAGGAG
		chr_1222-R	ACGGTCTGGCGGAGGTTG
14	*pks12*	chr_1219-F	ACCAACGGCGTCGGAGTG
		chr_1219-R	GCTTGTGCTGGAAGGCGTAG
15	*GdmG*	chr_1816-F1	AGTCGATGTGACCGAAGAAATT
		chr_1816-R1	GAAGGTGCCGATCTCCAAC
16	*GdmX*	chr_1803-F1	GCAGCATCACATCCTCAACA
		chr_1803-R1	GCGGAATTCCCAGTCGTAG
17	*rpoA*	chr_5758-F	GTCGCACGCTCCTCTCCTC
		chr_5758-R	ACGACGAGCTGCTTGATGTTG
18	*hrdB*	chr_6978-F1	GATTCCGCCAACCCAGTG
		chr_6978-R1	CTTCTGCGGCACTGACCA

Note: *rpoA* and *hrdB* were used as reference genes.

**Table 2:** Homozygous mutant genes.

Order	Mutant Gene	Name	Normal Base	Variant Base	Variation Type	Encoded Protein
1	chr-3081	*PpSQ1 00400*	C	T	nonsynonymous	dihydroxy-acid dehydratase
2	chr-1222	*pikAI*	CA	C	frameshift deletion	type I polyketide synthase
3	chr-1226	*pikAII*	C	CC	frameshift insertion	type I polyketide synthase

## Data Availability

Supplementary data to this article can be found online at Mendeley Data with DOI: 10.17632/yyz4kz7fxp.1.
